# Association mapping for morphological and physiological traits in *Populus simonii*

**DOI:** 10.1186/1471-2156-15-S1-S3

**Published:** 2014-06-20

**Authors:** Zunzheng Wei, Guanyu Zhang, Qingzhang Du, Jinfeng Zhang, Bailian Li, Deqiang Zhang

**Affiliations:** 1 National Engineering Laboratory for Tree Breeding, College of Biological Sciences and Technology, Beijing Forestry University, No. 35, Qinghua East Road, Beijing 100083, P. R. China; 2 Key Laboratory of Genetics and Breeding in Forest Trees and Ornamental Plants, College of Biological Sciences and Technology, Beijing Forestry University, No. 35, Qinghua East Road, Beijing 100083, P. R. China

**Keywords:** Association mapping, *Populus simonii*, Morphological traits, Physiological traits, SSR markers

## Abstract

**Background:**

To optimize marker-assisted selection programs, knowledge of the genetic architecture of phenotypic traits is very important for breeders. Generally, most phenotypes, e.g. morphological and physiological traits, are quantitatively inherited, and thus detection of the genes underlying variation for these traits is difficult. Association mapping based on linkage disequilibrium has recently become a powerful approach to map genes or quantitative trait loci (QTL) in plants.

**Results:**

In this study, association analysis using 20 simple sequence repeat (SSR) markers was performed to detect the marker loci linked to 13 morphological traits and 10 physiological traits in a wild *P. simonii *population that consisted of 528 individuals sampled from 16 sites along the Yellow River in China. Based on a model controlling for both population structure (Q) and relative kinship (K), three SSR markers (GCPM_616-1 in 31.2 Mb on LG I, GCPM_4055-2 in 5.7 Mb on LG XV, and GCPM_3142 of unknown location) were identified for seven traits. GCPM_616-1 was associated with five morphological traits (*R*^2 ^= 5.14-10.09%), whereas GCPM_3142 (15.03%) and GCPM_4055-2 (13.26%) were associated with one morphological trait and one physiological trait, respectively.

**Conclusions:**

The results suggest that this wild population is suitable for association mapping and the identified markers will be suitable for marker-assisted selection breeding or detection of target genes or QTL in the near future.

## Background

The development of fast-growing, highly adaptable and disease-resistant cultivars is a major focus in *Populus *breeding programs. To optimize marker-assisted selection programs, knowledge of the genetic architecture of phenotypic traits is very important for breeders. Generally, most phenotypes, e.g. morphological and physiological traits, are quantitatively inherited, and thus detection of the genes underlying variation for these traits is difficult. Mapping of quantitative trait loci (QTL) is a well-developed discipline that dissects the inheritance of complex traits into discrete Mendelian genetic factors [1]. Association mapping, also called linkage disequilibrium (LD) mapping, which directly studies statistical associations between genetic markers and phenotypes in natural populations, has recently regarded as promising approach to mapping QTL in crop plants. It can exploits all the recombination events that have occurred during the history of the population, allowing fine-scale QTL mapping [2-4]. Moreover, it bypasses the expense and shortens the duration of mapping studies by making the crossing cycles in population development unnecessary and enabling the mapping of many traits in one set of genotypes [2,5,6]. A concern about association mapping is that marker-trait associations may arise from confounding population structure, which may cause spurious correlations, leading to an elevated both Type I and II errors between molecular markers and traits of interest. However, estimates such as population structure (Q) and/or pair-wise kinship coefficients (K) were successfully applied to deal with the issue of false positives generated by population structure [2,3].

Generally, association mapping can be divided into genome-wide association mapping and candidate gene association mapping according to the scale (sample size), pre-known information (gene function and pathways), and purpose (questions to be addressed) of the studies [3]. Recently, the candidate gene method has been used to identify trait-marker relationships in poplar. In a pioneering association mapping study of a candidate region surrounding the *phytochrome B2 *(*phyB2*) locus in European Aspen (*Populus tremula*), two non-synonymous single nucleotide polymorphisms (SNPs) that independently associated with variation in the timing of bud set were identified and explained between 1.5 and 5% of the observed phenotypic variation in bud set [7]. Using the same panel, Ma et al [8] identified multi-SNPs from three genes in the photoperiod pathway (*PHYB2, LHY1*, and *LHY2*) associated with natural variation in growth cessation, which collectively explained 10-15% of the phenotypic variation. Li et al [9] conducted association analyses between leaf autumn senescence and SNPs derived from genes in the photoperiod pathway with naturally regenerated *P. tremula *populations. In addition, SNP- and haplotype-based association analysis in 426 *P. tomentosa *clones showed that nine SNPs and 12 haplotypes within UDP-glucuronate decarboxylase (*UXS*) were significantly associated with growth and wood property traits with 2.70% to 12.37% of the phenotypic variation [10]. However, whole-genome association studies have the advantage of enabling the entire genome to be assessed for trait-associated variants, rather than analyzing candidate genes [3-5].

Although more abundant SNP markers have been developed for poplar, genome-wide association mapping in poplar has rarely been attempted to date. This is largely because of the impracticality of genotyping large numbers of entries at the required number of SNP loci and the high development/detection cost. Compared to a SNP marker system, simple sequence repeat (SSR) markers remain an attractive marker system for genome-wide association mapping of poplar on account of their high variability, ubiquity, co-dominance, and easy availability. In addition, the most important factor is that a SSR marker system allows alignment to the black cottonwood (*P. trichocarpa*) genomic sequence, which provides information for comparative genomic studies of different species [11,12]. A large number of SSR primers for *Populus *have been designed from sequences that were randomly selected based on either library enrichment or shotgun sequencing strategies from various *Populus *species [13]. In addition, 148,428 SSR primers that amplified microsatellites consisting of repetitive motifs of 2-5 bp recently have been developed from unambiguously mapped sequence scaffolds of the *P. trichocarpa *genome [14].

*Populus simonii *is one of the most important native tree species in northern China and is widely distributed from Qinghai to the east coast in longitude and from the Heilongjiang River to the Yangtze River in latitude [15]. Owing to its large distribution range, excellent stress tolerance, rapid growth and regeneration ability, *P. simonii *plays an important and pioneering role in the stability and sustainability of forest ecosystems in northern China. In the present study, we performed association analysis of 20 SSR loci with 13 morphological traits and 10 physiological traits using 528 wild *P. simonii *individuals sampled from 16 sites along the Yellow River in China (Table S1 in Additional file 1). The major objectives were (1) to examine the population structure and familial relatedness of *P. simonii *and evaluate appropriate statistical models for association analysis, and (2) to identify the marker loci/QTL underlying the naturally occurring variation in the phenotypic traits.

## Results

### Phenotypic traits

As shown by the descriptive statistics presented in Table 1 extensive phenotypic variation was observed for all of the measured morphological and physiological traits in the *P. simonii *population. The lateral veins angle, which varied from 27.333° to 59.833° with an average of 43.359°, had the lowest change (2.2-fold), whereas leaf petiole length, which varied from 2.795 mm to 36.792 mm with an average of 10.136 mm, had the highest maximum change of 13.2-fold. Higher variation was observed for the 10 physiological traits (mean coefficient of variation 35.78%) than for the 13 morphological traits (24.06%). All traits were not normally distributed among the sampled individuals with two exceptions (lateral veins angle and ChlA content).

**Table 1:** Phenotypic variation for 13 morphological traits and 10 physiological traits in the wild *P. simonii *population

Traits	Min	Max	Mean	SD	CV	SW
Morphological traits						
LL (mm)	26.385	79.652	48.674	8.095	16.63%	*
LW(mm)	16.682	49.537	29.514	5.653	19.15%	**
LL/LW	1.255	2.977	1.698	0.190	11.21%	**
LA (cm^2^)	3.378	30.038	10.621	4.117	38.76%	**
LVL (mm)	10.157	37.648	21.664	4.093	18.89%	*
LVA (°)	27.333	59.833	43.359	4.749	10.95%	
LBA (°)	18.667	65.833	34.363	6.644	19.33%	**
LTN (n)	3.833	12.833	7.028	1.404	19.98%	**
LT(mm)	0.165	0.370	0.272	0.034	12.67%	*
LPL(mm)	2.795	36.792	10.136	6.603	65.14%	**
IL (mm)	7.313	36.758	21.480	5.899	27.46%	**
H (cm)	23.333	134.000	69.610	19.376	27.83%	**
D (cm)	0.090	0.360	0.186	0.046	24.83%	**
Physiological traits						
ChlA (mg/L)	2.773	15.443	9.282	2.450	26.39%	
ChlB(mg/L)	1.096	10.081	3.515	1.220	34.72%	**
ChlC(mg/L)	1.233	6.671	3.451	0.882	25.54%	**
SAR(μg/g)	10.938	64.964	24.607	10.842	44.06%	**
POD(U/g ▪ min)	0.262	1.111	0.779	0.139	17.81%	**
CAT(mg/ ▪ min)	0.621	3.992	1.533	0.814	53.09%	**
RCR (%)	8.528	34.096	16.438	3.999	24.33%	**
MDA(nmol/g)	5.081	40.276	12.243	6.608	53.98%	**
RRO(μg/g)	1.222	6.420	3.071	0.774	25.20%	**
PAL(U/g ▪ h)	0.094	0.521	0.172	0.091	52.71%	**

* *P *< 0.05, ** *P *< 0.01

### Population structure and relative kinship

**Figure 1:**
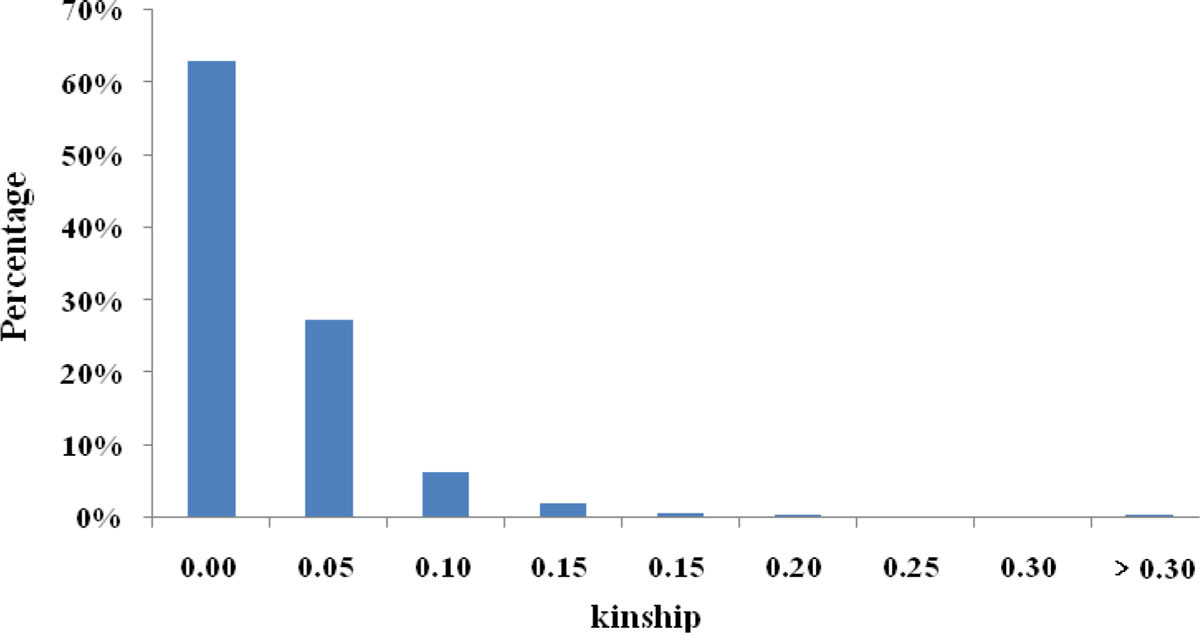
Distribution of the pair-wise relative kinship estimates between the 528 individuals of *P. simonii *based on data for 20 SSR markers.

Coupled with ΔK parameter computation [16], the percentage of admixture of each individual obtained for K = 3 [17] was used in the subsequent association analyses (Table S2 in Additional file 1). Relative kinship estimates based on the 20 SSR loci showed that 63.0% of the pair-wise kinship estimates equal to 0 suggests that almost two-thirds of the total pairs of accessions showed no relationship. As many as 99.6% of the relative kinship estimates were less than 0.30, which indicated that few individuals showed strong similarities, and most individuals were weakly related in this wild *P. simonii *population (Figure 1).

### Association mapping and allelic effect

Association mapping using 20 SSRs based on both the Q model and Q+K model was performed and is summarized in Table 2. For all 23 traits, a model that controlled for population structure and relative kinship performed significantly better than the model that merely controlled for population structure. Compared to the total number of significant markers identified with the Q model, the total number identified with the Q+K model was severely reduced by 199, 144 and 101 at *P *< 0.05, *P *< 0.01 and *P *< 0.001, respectively (Table 2). Despite correction for multiple tests in the Q model, the total associated marker number was still 14-fold lower in the Q+K model at qFDR < 0.05.

**Table 2:** Number of significant markers associated with 23 traits using two statistical models (Q and Q+K) at different significance levels

Tratis	Q				Q+K			
	
	*P *< 0.05	*P *< 0.01	*P *< 0.001	*P*_adj _< 0.05^a^	*P *< 0.05	*P *< 0.01	*P *< 0.001	q < 0.05^b^
Morphological traits								
LL	11	8	4	4	0	0	0	0
LW	9	7	4	4	0	0	0	0
LL/LW	6	3	3	1	1	1	0	1
LA	12	10	9	8	1	1	0	1
LVL	6	3	3	3	0	0	0	0
LVA	3	3	1	1	0	0	0	0
LBA	9	7	7	6	1	1	1	1
LTN	9	7	4	4	1	1	1	1
LT	13	12	7	7	0	0	0	0
LPL	15	13	12	12	1	1	1	1
IL	16	10	9	9	1	1	1	1
H	9	4	3	3	0	0	0	0
D	11	6	3	3	0	0	0	0
Physiological traits								
ChlA	12	10	7	8	2	0	0	0
ChlB	16	11	10	10	2	1	0	0
ChlC	14	11	8	9	2	0	0	0
SAR	4	3	1	1	1	0	0	0
POD	7	5	2	3	0	0	0	0
CAT	4	2	0	0	1	0	0	0
RCR	12	10	7	7	0	0	0	0
MDA	8	5	2	2	2	1	1	1
RRO	5	2	0	0	1	0	0	0
PAL	5	0	0	0	0	0	0	0
Total	216	152	106	105	17	8	5	7

^a ^The adjusted *P *values were obtained after a 50,000 permutation test, and these markers are shown in Table S4 in Additional file 1

^b ^The false discovery rate (DFR) or q values were obtained with the QVALUE R package, and these markers are shown in Tables 3

Using the Q model, the number of markers associated with morphological or physiological traits decreased with the increase in significance level, with a more than 30% decrease from *P *< 0.05 to *P *< 0.01, and almost 30% decrease from *P *< 0.01 to *P *< 0.001. The loci significant at the adjusted *P *values after a 50,000 permutation test were similar to those significant at the *P *< 0.001 level without the permutation test. The highest number of markers associated at *P*_adj _< 0.05 were identified for leaf petiole length (12 SSRs) followed by the ChlB content (10) and internode length (9), whereas the lowest number was found for CAT (0), RRO (0) and PAL (0). For the Q+K model, the number of associated markers for physiological traits (e.g. ChlA, ChlB, ChlC, SAR, MDA, and RRO) at *P *< 0.05, *P *< 0.01 and *P *< 0.001 was less stable than for morphological traits. At qFDR < 0.05, seven traits (LL/LW, LA, LBA, LTN, LPL, IL, and MDA) were only identified with one marker, respectively. However, more marker-trait associations were found for morphological traits than physiological traits overall regardless of the model.

Table 3 summarizes the significant markers and their phenotypic effects based on the Q+K model in the wild *P. simonii *population. For the six morphological traits, a total of six marker-trait associations were examined with two different markers. The SSR locus GCPM_616-1 on linkage group I was significant for five traits (LL/LW, LA, LBA, LTN, and IL) and explained a percentage of phenotypic variance that ranged from 5.14% for LA and 10.09% for LTN. In most instances, the presence of marker alleles 143 and 147 increased the phenotype value for all five morphological traits with the exception of the alleles 147 and 143 for LL/LW and LA separately. GCPM_3142 was significant only for LPL and explained the highest total phenotypic variance (15.03%), which indicated this SSR marker might be an important main-effect QTL that contributes to the leaf petiole length in *P. simonii*. After removal of rare alleles, six alleles were detected that showed a similar trend in increasing the leaf petiole length jointly or independently. Among the entire *P. simonii *panel, individuals carrying the allele 227 appeared to have a longer leaf petiole length compared to other alleles. For physiological traits, GCPM_4055-2 on linkage group XV was detected for MDA, with a higher proportion of the variation explained (13.26%). Two markers were associated with reduced malonaldehyde concentration but no significant allele effect was observed.

**Table 3:** Significant SSR markers and its phenotypic effects in the wild *P. simonii *population

Trait	Locus^a^	LG	Position (cM/Mb)	*P*	qFDR	*R* ^2 b^	Allele size (bp)	Allele effect
Morphological traits								
LL/LW	GCPM_616-1	I	-/31.2	0.0014	0.0051	0.0609	143	0.0002
							147	−0.0010
LA (cm^2^)	GCPM_616-1	I	-/31.2	0.007	0.0211	0.0514	143	−0.1017
							147	0.0268
LBA (°)	GCPM_616-1	I	-/31.2	7.18E-06	4.33E-05	0.0730	143	0.0514
							147	0.2174
LTN (n)	GCPM_616-1	I	-/31.2	7.26E-08	6.57E-07	0.1009	143	0.0626
							147	0.0607
IL (mm)	GCPM_616-1	I	-/31.2	9.75E-05	0.0004	0.0638	143	0.9784
							147	0.4136
LPL (mm)	GCPM_3142	-	-/-	1.24E-08	2.24E-07	0.1503	215	9.0707
							219	10.7675
							223	10.1148
							227	11.9544
							231	9.1360
							235	8.9211
Physiological traits								
MDA (nmol/g)	GCPM_4055-2	XV	-/5.7	7.14E-06	0.0003	0.1326	217	−3.5305
							229	−3.3007

^a ^Markers with a significant marker-trait association are reported at qFDR value < 0.05

^b ^*R*^2 ^indicates the percentage of the total variation explained by each locus

## Discussion

### Appropriate statistical model for association mapping

Correction for the confounding effects of population structure present in plant populations is essential for association mapping because the complex population structure may cause spurious correlations, which finally result in an elevated false-positive rate [4,5,18]. To reduce the probability of detecting false positive marker-trait associations, one major method, the structured association (SA) [3,18], has been suggested to account for population structure. In this method, the Q matrix estimated by the program Structure using a set of random markers is commonly incorporated in a general linear model (GLM) to test associations. However, Q matrix may not completely represent the population structure, although it can efficiently reduce the spurious associations. Yang et al [19] reported that structure program divides the panel into a few discrete populations, and the Q matrix only provides a rough dissection of population differentiation. Consequently, the K matrix [2] calculated using the SPAGeDi software package for familial relatedness has been broadened to combine with the Q matrix in a mixed linear model to improve the false positive detection rate, as described by Yu et al [2]. Additional studies have demonstrated that the Q+K model controlling for population structure and genetic relatedness, is better than the Q model [2,19,20]. The present results agreed with this finding, but with difference that the number of significant markers in the Q+K model was sharply reduced by more than 1100% at different *P *values compared with the Q model (Table 2). In fact, more than 60% of estimates of pairwise relatedness are around zero at K = 3, which means that the kinship relationships might not be important in affecting association mapping. However, the results show large effects between Q and Q+K models. The reason may be the *P. simonii *panel was derived from a mixture of individuals from 16 sites, which cause Hardy-Weinberg disequilibrium for single locus and LD for multiple loci. In addition, the lower number of SSR markers employed to estimate the kinship matrix may be another factor [20].

### Detection of phenotype-genotype association and additional perspectives

In the present study, genome-wide association mapping was applied to detect DNA markers tightly linked to agronomically and adaptively important traits. We detected three SSR markers, comprising GCPM_616-1, GCPM_4055-2 and GCPM_3142, for six morphological traits (LL/LW, LA, LBA, LTN, IL, and LPL) and one physiological trait (MDA) using the Q+K model. Of these markers, GCPM_616-1 was simultaneously associated with five morphological traits, which explained 5.14% to 10.09% of the phenotypic variance. Two possible explanations for this finding are closely linked genes or pleiotropy [21]. The other two markers explained more than 13% of the total phenotypic variance, which suggested that medium-effect QTL might be located near these SSR loci. The public release of the whole-genome sequence for *P. trichocarpa *Nisqually-1 enables alignment of the three SSR markers with the poplar genome sequence. The genetic position of each associated SSR marker is shown in Table 3. GCPM_616-1 and GCPM_4055-2 were observed on linkage groups I and XV, respectively, whereas GCPM_3142 was not examined. The physical position on the linkage group for GCPM_616-1 ranged from 31,165,745 to 31,165,891 bp, whereas the position of GCPM_4055-2 ranged from 5,665,052 to 5,665,276 bp.

To compare published QTL or SSR markers with those detected in the present study, we undertook a literature review for QTL reported for these traits in linkage mapping or association mapping studies. However, extremely limited information is available for this comparison in spite of the availability of a high-density SSR genetic map [22,25] derived from *P. trichocarpa*. The main reasons for this are probably because: (1) no common integrated genetic map that includes various types of molecular markers currently exists for *Populus *[14,21]; (2) the absence of conservative markers such as SSRs on genetic linkage maps for comparative mapping between *Populus *species [12,22]; and (3) the non-conformity of observed target traits for QTL mapping.

Understanding of the genetic bases underlying the naturally occurring genetic diversity and detection of genes or marker loci/QTL in the wild *P. simonii *population could assist breeders with MAS in plant breeding programs, thus making conventional breeding faster and more efficient. Association mapping is expected to achieve higher mapping resolution as it employs LD based on historical recombinations [4,5], which is supplemented with marker-assisted cloning or direct identification of the target gene against the genomic sequence [23,24]. Nevertheless, the power to detect and identify QTL or genes depends on the strength of the LD between the marker and the QTL or gene [4,5]. Currently, LD has been characterized to some extent in *P. trichocarpa *[25], *P. tremula *[7,26], *P. nigra *[27], and *P. balsamifera *[28]. Data from 100 short gene fragments or candidate gene regions of the above-mentioned *Populus *species showed that the LD level was expected to decay faster even with LD declining to negligible levels in less than several-hundred bases. Although LD is not constant either across the whole genome or along single chromosomes [3,4], we should confirm that the marker density surrounding the GCPM_616-1 and GCPM_4055-2 markers must be increased greatly so that the QTL or gene closely linked to traits of interest can be explored successfully.

## Materials and methods

### Plant materials and field trials

The 528 individuals sampled from 16 different localities along the Yellow River basin in China were used in this study [29]. One-year-old twigs collected from adult trees during fall and winter of 2007 were transferred to the greenhouse at Beijing Forestry University, where they were cut into 15 cm cuttings, placed in plant bags and stored with sand in a freezer (0°C) until planting in the following year. The cuttings were planted in a randomized complete block design with three replications at Xiaotangshan station in Beijing (39.9° E, 116.4° N) on 20 April, 2008. The distance between rows was 1.0 m, and the spacing between trees within a row was 0.8 m. Propagation effects such as cyclophysis and topophysis are known to have important impacts on growth of *Populus *and could lead to a slightly biased subset of clones included in the collection [30]. Therefore, we cut the stems above ground in December after all clones entered dormancy and started phenotypic measurements in the following year of growth in the field.

### Morphological and physiological traits characterization and data analysis

A total of 13 morphological traits and 10 physiological traits, were evaluated in 2009. Most morphological traits are relevant to leaf characteristics and evaluated following the methodology developed by He [31]. Three mature blades on the main stem of each clone in the field were selected to score leaf traits from July to September. The measured leaf traits comprised leaf length (LL), leaf width (LW), leaf area (LA), leaf thickness (LT), leaf petiole length (LPL), lateral veins length (LVL), lateral veins angle (LVA), leaf base angle (LBA) and number of leaf teeth (LTN). The ratios of leaf length to leaf width (LL/LW) were calculated for each measured leaf. In addition, the internode length (IL) was estimated by measuring the distance between two adjacent leaf scars along the stem and was repeated three times for every clone. Furthermore, the growth traits height (H) and diameter (D) were measured in November when the leaves were falling. The arithmetic mean of all individual morphological traits for three or nine measurements was used for the subsequent data analysis.

Physiological traits analyses were performed on an ultraviolet spectrophotometer (UV-2450/2550PC, Shimadzu) and an electrical conductivity meter (EC-4300, Suntex) using the methods described by Zhang et al [32] and Gao [33]. Leaves were sampled from clones of each genotype, then equally mixed and analyzed in the laboratory from August to October. The analyzed traits comprised the following: chlorophyll content (ChlA, ChlB, and ChlC), superoxide anion radicals content (SAR), peroxidase content (POD), catalase content (CAT), relative conductivity rate (RCR), malondialdehyde content (MDA), proline content (PRO) and phenylalanine ammonia lyase content (PAL). For all physiological traits, the same sample from each genotype was analyzed three times, and the average was used in the data analysis.

Descriptive statistical parameters such as the mean, standard deviation (SD), and coefficient of variation (CV) were determined for each phenotypic trait. Furthermore, the Shapiro-Wilk normality test (SW), measuring the data distribution of each trait, was carried out using the univariate procedure in SAS 9.0.

### SSR genotyping and physical position assignment

SSR markers were obtained from the International *Populus *Genome Consortium (IPGC, http://www.ornl.gov/sci/ipgc/ssr_resource.htm). Only 19 (14%) of 138 tested SSR markers showed polymorphisms across a randomly selected screening panel of 10 individuals [29]. The name, primer, LG, positions (cM or Mb), repeat motifs, allelic size and annealing temperature for the 19 polymorphic SSR markers are listed in Table S3 in Additional file 1. In addition, an EST-SSR primer within the coding region of the *Dehydration responsive element binding *(*DREB*) gene developed by Wei et al [34] was also used. Different PCR amplification conditions were used based on different annealing temperatures. DNA extraction, PCR amplification, and SSR genotyping followed previously described protocols [35]. Those alleles with a frequency fewer than 5% in the population were treated as rare alleles.

Assignment of a physical position to the SSR markers followed the method of Ranjan et al [36]. The SSR sequence information was first obtained from the PopGenIE In Silico PCR online resource (http://www.popgenie.org/tool/silico-pcr). Based on BLAST searches of the SSR primer nucleotide sequence against the genomic sequence, the physical position in the *Populus *genome was then assigned. In total, 14 markers were successfully assigned a physical position in the genome.

### Phenotype-genotype association analysis

Two covariate parameters, Q and K, were implemented to evaluate the effects of population structure and relative kinship, respectively, on phenotypic traits for marker-trait associations. The genetic structure (Q) among 528 clones was previously estimated by all 20 SSR markers (Table S4 in Additional file 1) with the model-based software Structure version 2.3.1 using a burn-in of 100,000 generations, run length of 5,000,000 generations, and 10 independent runs [18]. A model with admixture and correlated allele frequencies was chosen. The tested K values (equivalent to the number of subpopulations) ranged from one to 16. Based on the results of these runs, the ΔK parameter was estimated to identify the optimal number of clusters as described by Evanno et al [18]. The relative kinship (K) matrix was also calculated on the basis of 20 SSR loci using the method proposed by Ritland [37], which is implemented in the program SPAGeDi version 1.3 [38]. All negative values between individuals were set to 0 [2].

To correct for genetic structure and relatedness in this *P. simonii *population, two models were used and compared: (1) the Q model, which controlled for Q; and (2) the Q+K model, which controlled for both Q and K. The Q model was performed using a general linear model (GLM), whereas the Q+K model used a mixed linear model, with Tassel version 2.1 software [2]. In the Q model, 50,000 time permutations were employed for correction of multiple testing and markers with an adjusted *P*-value < 0.05 were regarded as significant. In the Q+K model, the default run parameters with the convergence criterion set at 1.0 × 10^−4 ^and the maximum number of iterations set at 200 were used. The qFDR value, an extension of the false discovery rate (FDR) method [39], was used to correct for multiple testing. The q values were calculated with the QVALUE R package using the smoother method [40]. Markers with DFR q < 0.05 were regarded as significant. Furthermore, to identify superior or inferior alleles that could be used or ignored in marker-assisted selection (MAS), allelic effects were estimated in comparison to the ''null allele'' (missing plus rare alleles) for each locus [41].

## Competing interests

The authors declared that they have no competing interests.

## Authors' contributions

Conceived and designed the experiments: DZ. Performed the experiments: ZW DZ, QD and GZ. Analyzed the data: ZW, DZ, QD, JZ and BL. Contributed reagents/materials/analysis tools: ZW and DZ. Wrote the paper: ZW, QD, and DZ.

## Funding

Publication of this work was supported by grants from: the Forestry Public Benefic Research Program (No. 201204306), and Program for Changjiang Scholars and Innovative Research Team in University (No. IRT13047), and Projects of the National Natural Science Foundation of China (No. 30600479, 30872042).

## Supplementary Material

Additional file 1 Table S1 Location, sampling site characteristics and sample sizes for all wild populations of *P. simonii*. Table S2 Estimates of the posterior probability of the data for a given K in wild populations of *P. simonii *. Table S3 SSR markers used for association mapping in wild populations of *P. simonii *. Table S4 Marker loci associated with morphological and physiological traits among the wild *P. simonii *populations based on the Q model Click here for file

## Notes

This article has been published as part of *BMC Genetics *Volume 15 Supplement 1, 2014: Selected articles from the International Symposium on Quantitative Genetics and Genomics of Woody Plants. The full contents of the supplement are available online at http://www.biomedcentral.com/bmcgenet/supplements/15/S1.
