# Supplementary material for: Association mapping for morphological and physiological traits in Populus simonii
Source: BMC Genet. 2014 Jun 20;15(Suppl 1):S3. doi: 10.1186/1471-2156-15-S1-S3 (PMC4118617; doi:10.1186/1471-2156-15-S1-S3)
Supplement: Additional file 1 — Table S1 Location, sampling site characteristics and sample sizes for all wild populations of P. simonii. Table S2 Estimates of the posterior probability of the data for a given K in wild populations of P. simonii . Table S3 SSR markers used for association mapping in wild populations of P. simonii . Table S4 Marker loci associated with morphological and physiological traits among the wild P. simonii populations based on the Q model [file 1471-2156-15-S1-S3-S1.zip › Additional file 1/Table S1.doc]

**Supplementary Table 1: Location, sampling site characteristics and sample sizes for all wild populations of *P. simonii***

| Basin | Province | Population | Latitude (E) | Longitude (N) | Sample size |
| --- | --- | --- | --- | --- | --- |
| North  -west | Qinghai | Huzhu | 102°07′48′′ | 36°33′36′′ | 14 |
|  |  | Qilian | 99°22′12′′ | 38°16′12′′ | 26 |
|  |  | Dulan | 98°03′00′′ | 36°10′12′′ | 35 |
| North  -east | Shanxi | Ningwu | 112°10′48′′ | 39°00′00′′ | 27 |
|  | Hebei | Zhangjiakou | 115°29′24′′ | 40°34′12′′ | 48 |
|  | Inner Mongolia | Naiman | 120°30′00′′ | 43°04′48′′ | 31 |
|  | Jilin | Tongyu | 123°03′00′′ | 44°32′24′′ | 43 |
|  | Liaoning | Chaoyang | 120°17′24′′ | 41°21′00′′ | 32 |
| South  -east | Shandong | Yishui | 118°22′12′′ | 35°43′12′′ | 7 |
| South | Shanxi | Fuxian | 109°13′12′′ | 35°35′24′′ | 45 |
|  |  | Luochuan | 109°29′24′′ | 35°27′00′′ | 45 |
|  | Gansu | Diebu | 103°02′24′′ | 34°15′00′′ | 44 |
|  | Sichuan | Kangding | 102°06′00′′ | 30°04′12′′ | 22 |
|  | Henan | Songxian | 112°01′12′′ | 33°34′12 ′′ | 36 |
|  |  | Yichuan | 112°17′24′′ | 34°12′36′′ | 35 |
|  |  | Luoning | 111°17′24′′ | 34°17′24′′ | 38 |
| Total | 11 | 16 |  |  | 528 |
