# Supplementary material for: Association mapping for morphological and physiological traits in Populus simonii
Source: BMC Genet. 2014 Jun 20;15(Suppl 1):S3. doi: 10.1186/1471-2156-15-S1-S3 (PMC4118617; doi:10.1186/1471-2156-15-S1-S3)
Supplement: Additional file 1 — Table S1 Location, sampling site characteristics and sample sizes for all wild populations of P. simonii. Table S2 Estimates of the posterior probability of the data for a given K in wild populations of P. simonii . Table S3 SSR markers used for association mapping in wild populations of P. simonii . Table S4 Marker loci associated with morphological and physiological traits among the wild P. simonii populations based on the Q model [file 1471-2156-15-S1-S3-S1.zip › Additional file 1/Table S2.doc]

***Supplementary Table 2 SSR markers used for association mapping in*** ***wild populations of*** P. simonii

| Marker name | Primer | Motif | LG | Position (cM) ^a^ | Position (Mb) ^b^ | Size | Tm (°C) |
| --- | --- | --- | --- | --- | --- | --- | --- |
| GCPM_2453-1 | F: ACACCAAGAGCTGTAGCATT  R: ACAACATggcctAACTCATC | ggt | [IX](http://genome.jgi-psf.org/cgi-bin/dispId?dbName=Poptr1_1&tableName=Genewise1&id=31215) | – | 6.0 | 200 | 47 |
| GCPM_2496-2 | F: GGATAATGAGTGTCAgaGGC  R: GCccTAGTAAAAGACCCTGT | gag | [X](http://genome.jgi-psf.org/cgi-bin/browserLoad?db=Poptr1&position=LG_X%3A10047224-10047442) | – | 10.0 | 215 | 47 |
| PMGC_2840 | F: TATATTGAGATCTCATCTAACAG  R: CAAAATTCAATTGTGTTGTAATC | GA | V | 130.1 | 14.5 | 228 | 41 |
| GCPM_2503-2 | F: CGGTGTTCATCATGACTATG  R: cgtaCAAGAgctGGTCtTtC | gag | [X](http://genome.jgi-psf.org/cgi-bin/browserLoad?db=Poptr1&position=LG_X%3A10047224-10047442) | – | 10.0 | 226 | 46 |
| ORPM_59 | F: TGCTAGTAACTGCGCATTGG  R: GATGTTTTTCGCACGCATTA | AT | XIV | 24.4 | – | 213 | 47 |
| GCPM_2570-1 | F: AACCCACTTCCTCTCTCTGT  R: GTGAGACTTCCGACTCGTAG | ct | I | – | 23.7 | 218 | 50 |
| ORPM_127 | F: TCAATGAGGGGTGCCATAAT  R: CTTTCCACTTTTGGCCCTTT | TG | IV | 49.1 | 7.5 | 200 | 48 |
| GCPM_2642-1 | F: TGAAATATTTAGCCATTATCCA  R: GGAAGGAGAATGAGAGGATT | ta | VI | – | 6.2 | 162 | 43 |
| GCPM_2870-1 | F: TATCTCTGTTGAGCAGCCTT  R: CTaGGtccgGAAGTTGATTT | ta | II | – | 22.2 | 227 | 47 |
| WPMS_7 | F: ACTAAGGAGAATTGTTGACTAC  R: TATCTGGTTTCCTCTTATGTG | GT | VI | 44.3 | – | 230 | 44 |
| GCPM_3142 | F: GAGGAAACGTTGGTCTGAT  R: GTCTCCACTGGACCAAAATA | aatt | – | – | – | 223 | 47 |
| ORPM_16 | F: GCAGAAACCACTGCTAGATGC  R: GCTTTGAGGAGGTGTGAGGA | CTT | XIII | 22.0 | – | 238 | 51 |
| PMGC_93 | F: ATCATGCGTTCGGCTACAGC  R: CTCAAACTCCAACTGTTATAAC | CTT | I | 148.6 | – | 350 | 46 |
| ORPM_206 | F: CCGTGGCCATTGACTCTTTA  R: GAACCCATTTGGTGCAAGAT | GCT | XIX/ XIII | 28.2 | 2.3/3.3 | 196 | 48 |
| GCPM_4055-2 | F: TACGgagaGGTGTTCTTgtt  R: TTGTAGGGGTAATcacAACC | atta | XV | – | 5.7 | 225 | 47 |
| ORPM_260 | F: TTCTAGTCCTGGCATAGCTTCA  R: CAGAGATTTGAATCGCAGCA | AAT | II | 23.7 | 18.3 | 220 | 47 |
| GCPM_616-1 | F: TTTTTCCCATTTGATAGCTT  R: AAGAAGTGGGAAAGGAACAT | at | i | – | 31.2 | 147 | 44 |
| WPMS_3 | F: TTTACATAGCATTTAGCCTTTAGA  R: TTATGATTTGGGGGTGTTATGGTA | GT | XII | 25.3 | 3.9 | 250 | 46 |
| ORPM_203 | F: CCACCAGGCATGAGATATGA  R: TCAAACCGAAAGGTCAACAA | TA | III | 61.4 | – | 209 | 48 |
| *DREB* | F: TGAGGTTGGAGCTCATGTT  R: AGTCAACCGGCCGTGGCTT | CAC | [VII](http://genome.jgi-psf.org/cgi-bin/browserLoad?db=Poptr1&position=LG_VII%3A8488683-8488858)/V | – | 8.5/5.3 | 165 | 58 |

^a^ The position (cM) of the SSR marker on the linkage group (LG) is in accordance with the International Populus Genome Consortium (IPGC; http://www.ornl.gov/sci/ipgc/ssr_resource.htm).

^b^ The physical position (Mb) of the SSR marker is based on the Populus Genome Integrative Explorer (PopGenIE) version 2.2 ( http://www.popgenie.org/tool/silico–pcr) and JGI *Populus trichocarpa* version 1.0 sequence release (http://genome.jgi–psf.org/Poptrl/Poptrl.home.html).
