# Supplementary material for: Association mapping for morphological and physiological traits in Populus simonii
Source: BMC Genet. 2014 Jun 20;15(Suppl 1):S3. doi: 10.1186/1471-2156-15-S1-S3 (PMC4118617; doi:10.1186/1471-2156-15-S1-S3)
Supplement: Additional file 1 — Table S1 Location, sampling site characteristics and sample sizes for all wild populations of P. simonii. Table S2 Estimates of the posterior probability of the data for a given K in wild populations of P. simonii . Table S3 SSR markers used for association mapping in wild populations of P. simonii . Table S4 Marker loci associated with morphological and physiological traits among the wild P. simonii populations based on the Q model [file 1471-2156-15-S1-S3-S1.zip › Additional file 1/Table S4.doc]

**Table S4 Marker loci associated with morphological and physiological traits among the wild *P. simonii* populations based on the Q model**

| Trait | Locus ^a^ | LG | Position (cM) | Position (Mb) | *P*_adj_ | *R*^2 b^ |
| --- | --- | --- | --- | --- | --- | --- |
| Morphological traits | | | | | | |
| LL | GCPM_2453-1 | [IX](http://genome.jgi-psf.org/cgi-bin/dispId?dbName=Poptr1_1&tableName=Genewise1&id=31215) | – | 6.0 | 2.00E-05 | 0.2368 |
|  | GCPM_2570-1 | I | – | 23.7 | 2.00E-05 | 0.273 |
|  | ORPM_16 | XIII | 22 | 22.0 | 2.00E-05 | 0.1731 |
|  | ORPM_203 | III | 61.4 | – | 0.002 | 0.2609 |
| LW | GCPM_2453-1 | [IX](http://genome.jgi-psf.org/cgi-bin/dispId?dbName=Poptr1_1&tableName=Genewise1&id=31215) | – | 6.0 | 2.00E-05 | 0.1703 |
|  | GCPM_2503-2 | [X](http://genome.jgi-psf.org/cgi-bin/dispId?dbName=Poptr1_1&tableName=Genewise1&id=31215) | – | 10.0 | 6.60E-04 | 0.2126 |
|  | ORPM_59 | XIV | 24.4 | – | 0.0036 | 0.2236 |
|  | GCPM_2570-1 | I | – | 23.7 | 0.0204 | 0.2344 |
| LL/LW | GCPM_2453-1 | [IX](http://genome.jgi-psf.org/cgi-bin/dispId?dbName=Poptr1_1&tableName=Genewise1&id=31215) | – | 6.0 | 2.00E-05 | 0.1268 |
| LA | GCPM_2453-1 | [IX](http://genome.jgi-psf.org/cgi-bin/dispId?dbName=Poptr1_1&tableName=Genewise1&id=31215) | – | 6.0 | 2.00E-05 | 0.203 |
|  | GCPM_2503-2 | [X](http://genome.jgi-psf.org/cgi-bin/dispId?dbName=Poptr1_1&tableName=Genewise1&id=31215) | – | 10.0 | 2.00E-05 | 0.2349 |
|  | ORPM_59 | XIV | 24.4 | – | 2.00E-05 | 0.269 |
|  | GCPM_2570-1 | I | – | 23.7 | 2.00E-05 | 0.3012 |
|  | ORPM_127 | IV | 49.1 | 7.5 | 0.0052 | 0.1157 |
|  | GCPM_2642-1 | VI | – | 6.2 | 0.0108 | 0.1123 |
|  | GCPM_2870-1 | II | – | 22.2 | 0.0186 | 0.203 |
|  | WPMS_7 | VI | 44.3 | – | 0.0477 | 0.0893 |
| LVL | GCPM_2453-1 | [IX](http://genome.jgi-psf.org/cgi-bin/dispId?dbName=Poptr1_1&tableName=Genewise1&id=31215) | – | 6.0 | 0.0017 | 0.1787 |
|  | GCPM_2503-2 | [X](http://genome.jgi-psf.org/cgi-bin/dispId?dbName=Poptr1_1&tableName=Genewise1&id=31215) | – | 10.0 | 0.0047 | 0.2286 |
|  | ORPM_59 | XIV | 24.4 | – | 0.0207 | 0.2124 |
| LVA | GCPM_2453-1 | [IX](http://genome.jgi-psf.org/cgi-bin/dispId?dbName=Poptr1_1&tableName=Genewise1&id=31215) | – | 6.0 | 0.0218 | 0.1069 |
| LBA | GCPM_2453-1 | [IX](http://genome.jgi-psf.org/cgi-bin/dispId?dbName=Poptr1_1&tableName=Genewise1&id=31215) | – | 6.0 | 2.00E-05 | 0.1465 |
|  | GCPM_2503-2 | [X](http://genome.jgi-psf.org/cgi-bin/dispId?dbName=Poptr1_1&tableName=Genewise1&id=31215) | – | 10.0 | 0.0012 | 0.1915 |
|  | ORPM_59 | XIV | 24.4 | – | 0.0012 | 0.2172 |
|  | GCPM_2570-1 | I | – | 23.7 | 0.0103 | 0.1838 |
|  | ORPM_127 | IV | 49.1 | 7.5 | 0.0138 | 0.0657 |
|  | GCPM_2642-1 | VI | – | 6.2 | 0.0207 | 0.0907 |
| LTN | GCPM_2453-1 | [IX](http://genome.jgi-psf.org/cgi-bin/dispId?dbName=Poptr1_1&tableName=Genewise1&id=31215) | – | 6.0 | 2.00E-05 | 0.2128 |
|  | GCPM_2503-2 | [X](http://genome.jgi-psf.org/cgi-bin/dispId?dbName=Poptr1_1&tableName=Genewise1&id=31215) | – | 10.0 | 2.00E-05 | 0.2281 |
|  | ORPM_59 | XIV | 24.4 | – | 2.00E-05 | 0.2425 |
|  | GCPM_2570-1 | I | – | 23.7 | 0.0452 | 0.1895 |
| LT | GCPM_2453-1 | [IX](http://genome.jgi-psf.org/cgi-bin/dispId?dbName=Poptr1_1&tableName=Genewise1&id=31215) | – | 6.0 | 2.00E-05 | 0.2721 |
|  | GCPM_2503-2 | [X](http://genome.jgi-psf.org/cgi-bin/dispId?dbName=Poptr1_1&tableName=Genewise1&id=31215) | – | 10.0 | 2.00E-05 | 0.2234 |
|  | ORPM_59 | XIV | 24.4 | – | 2.00E-05 | 0.2209 |
|  | GCPM_2570-1 | I | – | 23.7 | 2.00E-05 | 0.2755 |
|  | ORPM_127 | IV | 49.1 | 7.5 | 0.0046 | 0.0792 |
|  | GCPM_2642-1 | VI | – | 6.2 | 0.0112 | 0.1353 |
|  | GCPM_2870-1 | II | – | 22.2 | 0.0199 | 0.1526 |
| LPL | GCPM_2453-1 | [IX](http://genome.jgi-psf.org/cgi-bin/dispId?dbName=Poptr1_1&tableName=Genewise1&id=31215) | – | 6.0 | 2.00E-05 | 0.2232 |
|  | GCPM_2503-2 | [X](http://genome.jgi-psf.org/cgi-bin/dispId?dbName=Poptr1_1&tableName=Genewise1&id=31215) | – | 10.0 | 2.00E-05 | 0.2134 |
|  | ORPM_59 | XIV | 24.4 | – | 2.00E-05 | 0.1812 |
|  | GCPM_2570-1 | I | – | 23.7 | 2.00E-05 | 0.4487 |
|  | ORPM_127 | IV | 49.1 | 7.5 | 2.00E-05 | 0.136 |
|  | GCPM_2642-1 | VI | – | 6.2 | 2.00E-05 | 0.1805 |
|  | GCPM_2870-1 | II | – | 22.2 | 2.00E-05 | 0.1298 |
|  | WPMS_7 | VI | 44.3 | – | 2.00E-05 | 0.1314 |
|  | GCPM_3142 | – | – | – | 2.00E-05 | 0.2252 |
|  | ORPM_16 | XIII | 22 | 22.0 | 0.001 | 0.0612 |
|  | PMGC_93 | I | 148.6 | – | 0.0011 | 0.1061 |
|  | GCPM_4055-2 | XV | – | 5.7 | 0.0149 | 0.0845 |
| IL | GCPM_2453-1 | [IX](http://genome.jgi-psf.org/cgi-bin/dispId?dbName=Poptr1_1&tableName=Genewise1&id=31215) | – | 6.0 | 2.00E-05 | 0.3039 |
|  | GCPM_2503-2 | [X](http://genome.jgi-psf.org/cgi-bin/dispId?dbName=Poptr1_1&tableName=Genewise1&id=31215) | – | 10.0 | 2.00E-05 | 0.2363 |
|  | ORPM_59 | XIV | 24.4 | – | 2.00E-05 | 0.2388 |
|  | GCPM_2570-1 | I | – | 23.7 | 2.00E-05 | 0.3003 |
|  | ORPM_127 | IV | 49.1 | 7.5 | 2.00E-05 | 0.168 |
|  | GCPM_2642-1 | VI | – | 6.2 | 2.00E-05 | 0.1389 |
|  | GCPM_2870-1 | II | – | 22.2 | 2.00E-05 | 0.14 |
|  | WPMS_7 | VI | 44.3 | – | 0.0022 | 0.1694 |
|  | GCPM_3142 | – | – | – | 0.0041 | 0.1263 |
| H | GCPM_2453-1 | [IX](http://genome.jgi-psf.org/cgi-bin/dispId?dbName=Poptr1_1&tableName=Genewise1&id=31215) | – | 6.0 | 2.00E-05 | 0.1842 |
|  | GCPM_2503-2 | [X](http://genome.jgi-psf.org/cgi-bin/dispId?dbName=Poptr1_1&tableName=Genewise1&id=31215) | – | 10.0 | 0.0115 | 0.193 |
|  | ORPM_59 | XIV | 24.4 | – | 0.0142 | 0.1808 |
| D | GCPM_2453-1 | [IX](http://genome.jgi-psf.org/cgi-bin/dispId?dbName=Poptr1_1&tableName=Genewise1&id=31215) | – | 6.0 | 2.00E-05 | 0.1699 |
|  | GCPM_2503-2 | [X](http://genome.jgi-psf.org/cgi-bin/dispId?dbName=Poptr1_1&tableName=Genewise1&id=31215) | – | 10.0 | 2.00E-05 | 0.2162 |
|  | ORPM_59 | XIV | 24.4 | – | 0.0137 | 0.1932 |
|  | GCPM_2570-1 | I | – | 23.7 | 0.0802 | 0.2442 |
| Physiological traits | | | | | | |
| ChlA | GCPM_2453-1 | [IX](http://genome.jgi-psf.org/cgi-bin/dispId?dbName=Poptr1_1&tableName=Genewise1&id=31215) | – | 6.0 | 2.20E-04 | 0.2334 |
|  | GCPM_2503-2 | [X](http://genome.jgi-psf.org/cgi-bin/dispId?dbName=Poptr1_1&tableName=Genewise1&id=31215) | – | 10.0 | 2.00E-05 | 0.2982 |
|  | ORPM_59 | XIV | 24.4 | – | 2.00E-05 | 0.2914 |
|  | GCPM_2570-1 | I | – | 23.7 | 2.60E-04 | 0.2866 |
|  | ORPM_127 | IV | 49.1 | 7.5 | 4.60E-04 | 0.1877 |
|  | GCPM_2642-1 | VI | – | 6.2 | 0.0023 | 0.2244 |
|  | GCPM_2870-1 | II | – | 22.2 | 0.0125 | 0.1841 |
|  | WPMS_7 | VI | 44.3 | – | 0.0364 | 0.0966 |
| ChlB | GCPM_2453-1 | [IX](http://genome.jgi-psf.org/cgi-bin/dispId?dbName=Poptr1_1&tableName=Genewise1&id=31215) | – | 6.0 | 2.00E-05 | 0.2394 |
|  | GCPM_2503-2 | [X](http://genome.jgi-psf.org/cgi-bin/dispId?dbName=Poptr1_1&tableName=Genewise1&id=31215) | – | 10.0 | 2.00E-05 | 0.3367 |
|  | ORPM_59 | XIV | 24.4 | – | 2.00E-05 | 0.2547 |
|  | GCPM_2570-1 | I | – | 23.7 | 2.00E-05 | 0.2842 |
|  | ORPM_127 | IV | 49.1 | 7.5 | 2.00E-05 | 0.1742 |
|  | GCPM_2642-1 | VI | – | 6.2 | 0.0016 | 0.1723 |
|  | GCPM_2870-1 | II | – | 22.2 | 0.0127 | 0.2541 |
|  | WPMS_7 | VI | 44.3 | – | 0.0208 | 0.0705 |
|  | GCPM_3142 | – | – | – | 0.0214 | 0.114 |
|  | ORPM_16 | XIII | 22 | 22.0 | 0.0429 | 0.1878 |
| ChlC | GCPM_2453-1 | [IX](http://genome.jgi-psf.org/cgi-bin/dispId?dbName=Poptr1_1&tableName=Genewise1&id=31215) | – | 6.0 | 2.00E-05 | 0.2519 |
|  | GCPM_2503-2 | [X](http://genome.jgi-psf.org/cgi-bin/dispId?dbName=Poptr1_1&tableName=Genewise1&id=31215) | – | 10.0 | 2.00E-05 | 0.3036 |
|  | ORPM_59 | XIV | 24.4 | – | 2.00E-05 | 0.2865 |
|  | GCPM_2570-1 | I | – | 23.7 | 2.00E-05 | 0.3192 |
|  | ORPM_127 | IV | 49.1 | 7.5 | 2.00E-05 | 0.1877 |
|  | GCPM_2642-1 | VI | – | 6.2 | 9.20E-04 | 0.229 |
|  | GCPM_2870-1 | II | – | 22.2 | 0.0011 | 0.2042 |
|  | WPMS_7 | VI | 44.3 | – | 0.002 | 0.0969 |
|  | GCPM_3142 | – | – | – | 0.0454 | 0.1294 |
| SAR | GCPM_2453-1 | [IX](http://genome.jgi-psf.org/cgi-bin/dispId?dbName=Poptr1_1&tableName=Genewise1&id=31215) | – | 6.0 | 0.0042 | 0.1791 |
| POD | GCPM_2453-1 | [IX](http://genome.jgi-psf.org/cgi-bin/dispId?dbName=Poptr1_1&tableName=Genewise1&id=31215) | – | 6.0 | 0.0066 | 0.2031 |
|  | GCPM_2503-2 | [X](http://genome.jgi-psf.org/cgi-bin/dispId?dbName=Poptr1_1&tableName=Genewise1&id=31215) | – | 10.0 | 0.0193 | 0.1894 |
|  | ORPM_59 | XIV | 24.4 | – | 0.0343 | 0.308 |
| RCR | GCPM_2453-1 | [IX](http://genome.jgi-psf.org/cgi-bin/dispId?dbName=Poptr1_1&tableName=Genewise1&id=31215) | – | 6.0 | 2.00E-05 | 0.2446 |
|  | GCPM_2503-2 | [X](http://genome.jgi-psf.org/cgi-bin/dispId?dbName=Poptr1_1&tableName=Genewise1&id=31215) | – | 10.0 | 2.00E-05 | 0.359 |
|  | ORPM_59 | XIV | 24.4 | – | 2.00E-05 | 0.2471 |
|  | GCPM_2570-1 | I | – | 23.7 | 2.00E-05 | 0.2099 |
|  | ORPM_127 | IV | 49.1 | 7.5 | 2.00E-05 | 0.2066 |
|  | GCPM_2642-1 | VI | – | 6.2 | 0.0014 | 0.141 |
|  | GCPM_2870-1 | II | – | 22.2 | 0.0252 | 0.3685 |
| MDA | GCPM_2453-1 | [IX](http://genome.jgi-psf.org/cgi-bin/dispId?dbName=Poptr1_1&tableName=Genewise1&id=31215) | – | 6.0 | 2.80E-04 | 0.2356 |
|  | GCPM_2503-2 | [X](http://genome.jgi-psf.org/cgi-bin/dispId?dbName=Poptr1_1&tableName=Genewise1&id=31215) | – | 10.0 | 2.00E-05 | 0.2068 |
|  | ORPM_59 | XIV | 24.4 | – | 0.0379 | 0.2102 |
|  | GCPM_2570-1 | I | – | 23.7 | 0.0428 | 0.2048 |

^a^ Markers with *P*_adj_ value < 0.05 were reported as significant

^b^ *R*^2^ indicates the percentage of the total variation explained
